# Distinct Expression Patterns Of Causative Genes Responsible For Hereditary Progressive Hearing Loss In Non-Human Primate Cochlea

**DOI:** 10.1038/srep22250

**Published:** 2016-02-26

**Authors:** Makoto Hosoya, Masato Fujioka, Kaoru Ogawa, Hideyuki Okano

**Affiliations:** 1 Keio University School of Medicine, Department of Otorhinolaryngology, Head and Neck Surgery, 35 Shinanomachi Shinjyuku-ku Tokyo, 160-8582, Japan; 2 Keio University School of Medicine, Department of Physiology, 35 Shinanomachi Shinjyuku-ku Tokyo, 160-8582, Japan

## Abstract

Hearing impairment is the most frequent sensory deficit in humans. Deafness genes, which harbor pathogenic mutations that have been identified in families with hereditary hearing loss, are commonly expressed in the auditory end organ or the cochlea and may contribute to normal hearing function, yet some of the mouse models carrying these mutations fail to recapitulate the hearing loss phenotype. In this study, we find that distinct expression patterns of those deafness genes in the cochlea of a non-human primate, the common marmoset (*Callithrix jacchus*). We examined 20 genes whose expression in the cochlea has already been reported. The deafness genes *GJB3, CRYM, GRHL2, DFNA5*, and *ATP6B1* were expressed in marmoset cochleae in patterns different from those in mouse cochleae. Of note, all those genes are causative for progressive hearing loss in humans, but not in mice. The other tested genes, including the deafness gene *COCH*, in which mutation recapitulates deafness in mice, were expressed in a similar manner in both species. The result suggests that the discrepancy in the expression between rodents and primates may account for the phenotypic difference. This limitation of the rodent models can be bypassed by using non-human primate models such as the marmoset.

Deafness is one of the most common types of congenital impairment, and at least half of which are caused by hereditary mutations. In some patients, hearing loss is progressive, with hearing loss developing gradually during childhood or youth after the acquisition of speech abilities. In these post-lingual hearing loss cases, a hearing aid or cochlea implant is effective because the auditory cortex has already developed^1^. Therefore, the prevention of progressive hearing loss would be one of the most promising therapeutics for long-term hearing ability.

As of 2015, the genes responsible for hearing loss have been identified in 99 autosomal recessive^2^, 67 autosomal dominant^3^, and six X-chromosomal recessive loci^4^. While mouse models for the vast majority of deafness genes recapitulate the human phenotype, in a few cases mouse models for human deafness did not result in hearing loss^5^,^6^,^7^. Especiallycin such cases, detailed mechanisms by which they cause hearing loss are not fully understood because of the difficulty in obtaining human cochleae suitable for pathological analyses. First, the number of human subjects during the development in hearing impairment is limited because it is non-lethal symptom. Second, there are technical difficulties of fixation and decalcification with the immunoreactivity retained. Third, the cochlea atrophies during normal aging^8^ and age-matching healthy controls can be difficult. Finally, there are differences in the physiology of individual cell types and/or proteins in the cochlea between humans and rodents (including mice, rats, guinea pigs, and gerbils), which are frequently used as animal models for examining gene expression patterns and determining the pathophysiology. Indeed, frequently such models recapitulate the human pathophysiology. However, some rodent models for hereditary hearing loss fail to recapitulate the phenotypes of human patients^5^,^7^. Such discrepancies can be accounted by a biological difference between species, but the details remain unknown.

Therefore, we wanted to use non-human primates to investigate the anatomy of hearing organs and the pathophysiology of hearing loss. In particular, we were interested in a small New World monkey species, the common marmoset (*Callithrix jacchus*), which exhibits more human-associated traits than Old World monkeys, including a richer variety of vocalizations and verbal communication^9^. Furthermore, the marmoset’s hearing range overlaps with that of humans^10^, suggesting the significance of the marmoset as a model for hearing research. Because genetic modification is now possible in the common marmoset^11^, we can use this species as a non-human primate model for the investigation of the detailed pathogenic mechanisms of hearing loss.

In this study, we established a successful immunohistochemical approach for the common marmoset and examined the expression patterns of 20 genes in the inner ear, genes whose expression has already been investigated in rodent or human cochleae (Fig. 1a). The genes tested include well-known genes causal for hereditary progressive hearing loss such as *GJB3* (CX31, DFNA2B, DFNB91), *CRYM*, *GRHL2* (DFNA28), *DFNA5*, and *ATP6B1* (distal renal tubular acidosis (dRTA) with hearing loss), all of whose pathophysiology remains unknown because their rodent models did not recapitulate the deafness phenotype. We found that these genes had distinct expression patterns, in which the proteins were distributed in the cochlea differently in primates and rodents. We also examined the expression profiles of COCH (*DFNA9*) and CONNEXIN26 (*CX26*, *DFNA3A*, *DFNB1A*), which have mouse models that recapitulate the deafness phenotype. We found that those proteins were expressed in a pattern similar to that reported for rodents.

Taken together, our results suggest that the discrepancy of the expression between rodents and primates accounts for the phenotypic difference between them, and that the limitations of rodent models in researching human hereditary hearing loss could be bypassed by using non-human primate models such as the common marmoset.

## Results

### Immunohistochemical analysis of marmoset cochleae

**Figure 1:**
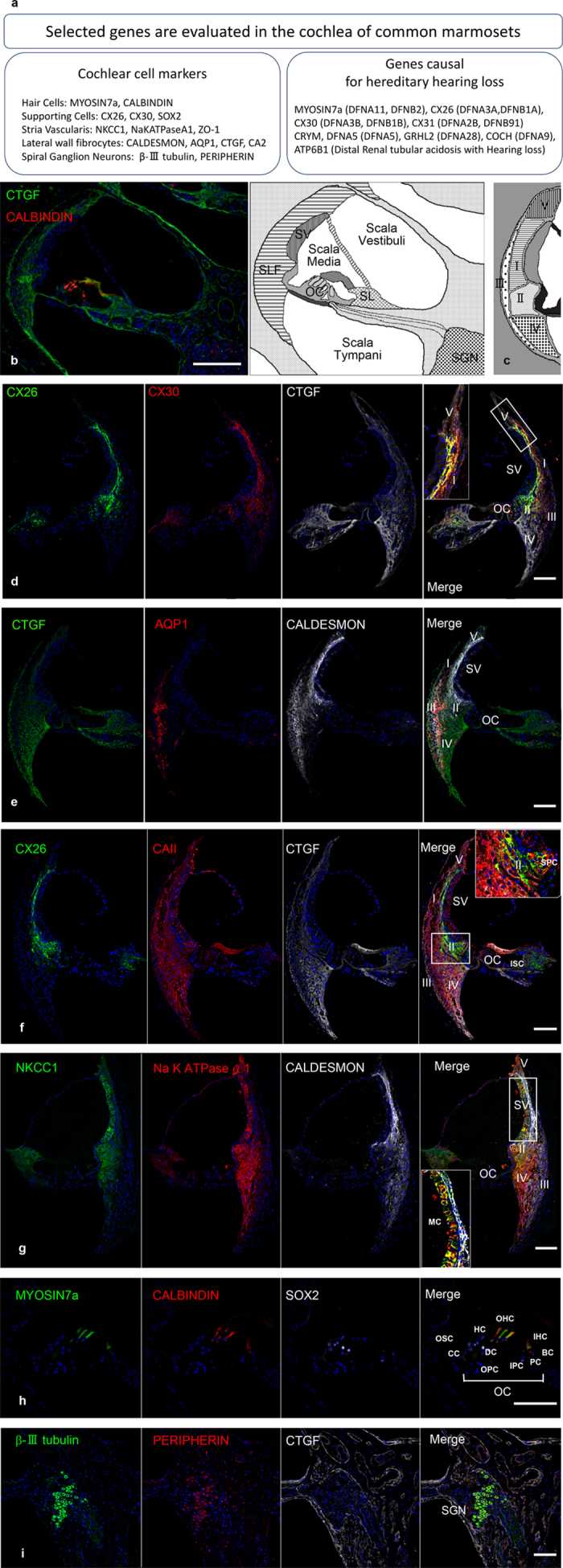
Expression of conventional markers in the cochlea of the common marmoset (**a**) Selected genes are evaluated in the cochlea of common marmosets. We selected 14 cochlear cell markers and nine genes causal for hereditary hearing loss. Antibodies specific to them were used in this study. (**b**) A cross-sectional view of the common marmoset cochlea duct under low magnification (left). The corresponding schema with anatomical landmarks is shown (right). (**c**) Schema for the subtypes of lateral wall fibrocytes. (**d–g**) The expression patterns of conventional markers in the lateral wall. CX26, CX30, CTGF, AQP1, CALDESMON, NKCC1, and Na^+^/K^+^ ATPase α1 expression were all observed in the cochleae, as well as previously in rodent. High magnification images were shown in the most right panels of (**f,g**). (**h**) Expression patterns of conventional markers in OC. MYOSIN 7a and CALBINDIN expression was observed in three rows of outer hair cells and one row of inner hair cells. SOX2 expression was observed in inner pillar cells, outer pillar cells, Deiters’ cells, and Hensen’s cells. (**i**) Expression pattern of conventional markers in SGNs. Expression of βIII TUBULIN and PERIPHERIN was observed in marmoset SGNs. The nuclei were counterstained with Hoechst (blue). Scale bar: 200 μm in b, 100 μm in (**c–i**). Basal turns in (**d–g,i**), apical turn in h. SV: stria vascularis, SLF: spiral ligament fibrocytes (I-V: Type I-V), OC: organ of Corti, SL: spiral limbus, SGN: spiral ganglion neurons, MC: Marginal cells, SPC: Spiral prominence cells, ISC: Inner sulcus cells, OSC: Outer sulcus cells, CC: Claudius cells, HC: Hensen’s cells, DC: Deiters’ cells, IPC: inner pillar cells, OPC: outer pillar cells, PC: inner phalangeal cells, BC: border cells, OHC: Outer hair cells, IHC: Inner hair cells.

Few studies^12^,^13^ have been conducted of the morphology of the temporal bone in the common marmoset, which have the cochlea with 2 and 3/4 turns (Supplementary Fig. S1), and no experiments using immunohistochemistry have been reported. To fully optimize the final protocol for histochemical analyses, we tested several methods including modifications of local perfusion methods for fixations, decalcification periods, thickness of frozen sections and antigen retrievals. The smaller size of the temporal bone in marmosets enabled us to investigate the histopathology using an easier and shorter decalcification process without excess fixation, resulting in the preservation of structures and immunoreactivity. Whereas it takes 3–5 months on average to decalcify human temporal bones^14^,^15^, it took 3–4 weeks to sufficiently decalcify the marmoset bones. As shown in Fig. 1b, we successfully immunostained samples using the optimized frozen section methods. Notably, the temporal bone sections showed that the general morphology of the marmosets’ cochleae is similar to that of humans.

### Expression of conventional markers in the cochlea

We next collected basic information on the marmoset cochlea using immunostaining. First, we examined several lateral wall cell markers, CONNEXIN 26 (CX26), CX30, CTGF, AQUAPORIN 1 (AQP1), CALDESMON, CARBONIC ANHYDRASE II (CAII), Na-K-Cl COTRANSPORTER 1 (NKCC1), Na^+^/K^+^ ATPase α1, and CTGF. The lateral wall of the cochlea is composed of type I–V lateral wall fibrocytes and three layers of cells in the stria vascularis (marginal cells, intermediate cells, and basal cells). The cell types of the lateral wall fibrocytes are defined by their spatial distribution and structure^16^,^17^ (Fig. 1c). Immunoreactivity for CX26 was observed in type I and II fibrocytes, the basal cells of the stria vascularis, spiral prominence cells, outer sulcus cells, supporting cells, and spiral limbus cells. CX30 immunoreactivity was observed in type I, II, III, and V fibrocytes, spiral prominence cells, outer sulcus cells, supporting cells, and spiral limbus cells. AQP1 immunoreactivity was observed specifically in type III fibrocytes. CALDESMON immunoreactivity was observed in type I and III fibrocytes. CAII immunoreactivity was observed in type I, III, and IV fibrocytes and the intermediate cells of the stria vascularis. NKCC1 immunoreactivity was observed in type II and IV fibrocytes, marginal cells, and spiral limbus cells. Na^+^/K^+^ ATPase α1 immunoreactivity was observed in type II, IV, and V fibrocytes and marginal cells. CTGF immunoreactivity was observed in type I, II, III, IV, and V fibrocytes, the basilar membrane, and the spiral limbus (Fig. 1d–g).

In the organ of Corti, immunoreactivity for MYOSIN 7a (MYO7a) and CALBINDIN was observed in three rows of outer hair cells and one row of inner hair cells. SOX2, a well-established supporting cell marker, was observed in some supporting cells: Hensen’s cells, Deiters’ cells, inner pillar cells, outer pillar cells, inner phalangeal cells, and border cells (Fig. 1h). In spiral ganglion neurons (SGNs), immunoreactivity for β-III TUBULIN and PERIPHERIN was observed.

The expression of CX26, CX30, CTGF, AQP1, CALDESMON, CAII, NKCC1, Na^+^/K^+^ ATPase α1, MYO7a, SOX2, and β-III TUBULIN in the cochlea of common marmosets was similar to that previously reported in mouse and other rodents. On the other hand, two proteins, CTGF and PERIPHERIN, were expressed differently between marmosets and rodents: CTGF expression was observed in all types of fibrocytes in the common marmoset, whereas its expression in mice is limited to type IV fibrocytes^18^. PERIPHERIN expression was observed in most SGNs, and its expression pattern was different from that of rodents, but quite similar to that of humans^19^. The gross morphology of the cross section and AQP1 expression suggest that, in the common marmoset, several rows of type III fibrocytes are distributed in the outer part of the lateral wall, but not in the bone capsule, whereas in the rodent, the cells are distributed in a single border layer next to the bone^20^. This distribution pattern of type I fibrocytes is similar to that of human temporal bones^21^.

### Expression pattern of the deafness gene GJB3 (CX31, DFNA2B/DFNB91)

**Figure 2:**
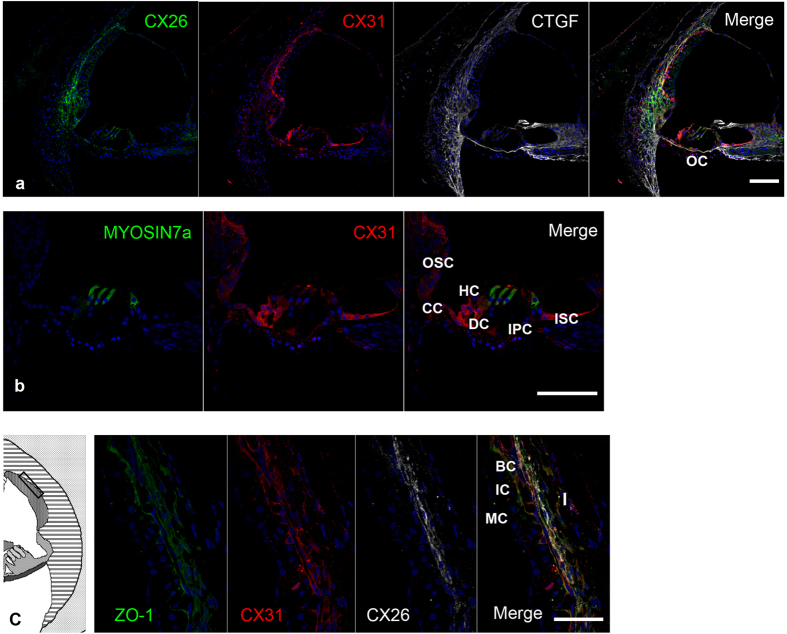
CONNEXIN 31 expression in the cochlea of the common marmoset (**a**) CX31 expression was observed in the type I fibrocytes of the spiral ligaments, the basal cells of the stria vascularis, Reissner’s membrane, and supporting cells. (**b**) CX31 expression was observed in inner sulcus cells, Hensen’s cells, Claudius cells, and outer sulcus cells. (**c**) Cx31 expression was observed in the stria vascularis and type I fibrocytes. CX31 expression was observed in the ZO-1-positive basal cells of the stria vascularis and type I fibrocytes. In type I fibrocytes, CX31 expression partially overlapped with CX26 expression. The nuclei were counterstained with Hoechst (blue). Scale bar: 100 μm in (**a,b**), 50 μm in (**c**). Basal turns in (**a,c**), middle turn in b. OC: organ of Corti, ISC: Inner sulcus cells, OSC: Outer sulcus cells, CC: Claudius cells, HC: Hensen’s cells, DC: Deiters’ cells, IPC: inner pillar cells, I: spiral ligament fibrocytes Type I, MC: Marginal cells, IC: Intermediate cells, BC: Basal cells.

CX31, also known as gap junction beta-3 protein (GJB3) or DFNA2B/DFNB91, is a gap junction protein, and in humans it is encoded by the *GJB3* gene^22^. Its expression in mouse cochlea is limited to the lateral wall and spiral limbus^23^. In marmoset cochlea, CX31 immunoreactivity was observed in the type I fibrocytes of the spiral ligaments, the basal cells of the stria vascularis, Reissner’s membrane, and supporting cells (inner sulcus cells, Hensen’s cells, Claudius cells, and outer sulcus cells) (Fig. 2a,b). Immunoreactivity for CX31 in other supporting cells was not observed, specifically in Deiters’ cells, outer pillar cells, inner pillar cells, and hair cells. Immunoreactivity for CX31 in type I fibrocytes was partially co-localized with that of CX26. In the stria vascularis, immunoreactivity for CX31 was observed in basal cells and co-localized with ZO-1 (Fig. 2c).

### Expression pattern of the deafness gene CRYM

**Figure 3:**
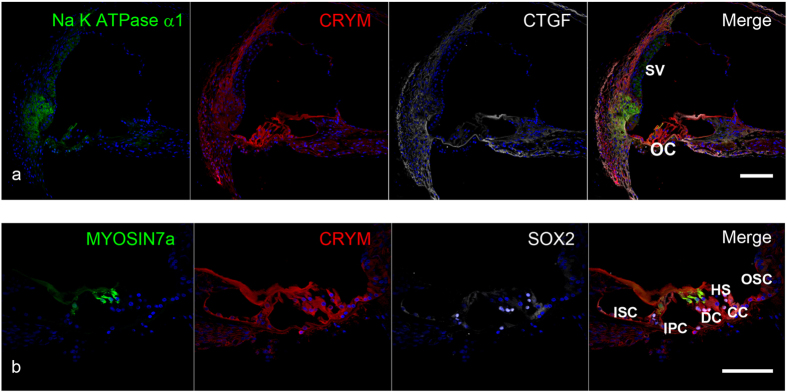
CRYM expression in the cochlea of the common marmoset (**a**) CRYM expression was observed in the lateral wall spiral ligament, both inner and outer hair cells, supporting cells, and the spiral limbus. (**b**) CRYM expression in the supporting cells was broadly observed between the inner sulcus and outer sulcus cells. CRYM expression was also observed in inner and outer hair cells. The nuclei were counterstained with Hoechst (blue). Scale bar: 100 μm. Middle turns in (**a,b**). SV: stria vascularis, OC: organ of Corti, ISC: Inner sulcus cells, OSC: Outer sulcus cells, CC: Claudius cells, HC: Hensen’s cells, DC: Deiters’ cells, IPC: inner pillar cells.

CRYM (mu-crystallin homolog), also known as NADP-regulated thyroid-hormone-binding protein (THBP), is a crystallin structural protein encoded by the *CRYM* gene. In humans, CRYM is expressed exclusively in the inner ear, as shown by a cDNA microarray analysis of gene expression^24^. In mice it exhibits limited expression in the lateral wall and the spiral limbus^24^,^25^. We found that the marmoset had broader expression, not only in the lateral wall spiral ligament and the spiral limbus, but also in both inner and outer hair cells, supporting cells, (Fig. 3a). Expression was found in all types of the supporting cells between the inner sulcus and outer sulcus cell (Fig. 3b).

### Expression pattern of the deafness gene GRHL2 (DFNA28)

**Figure 4:**
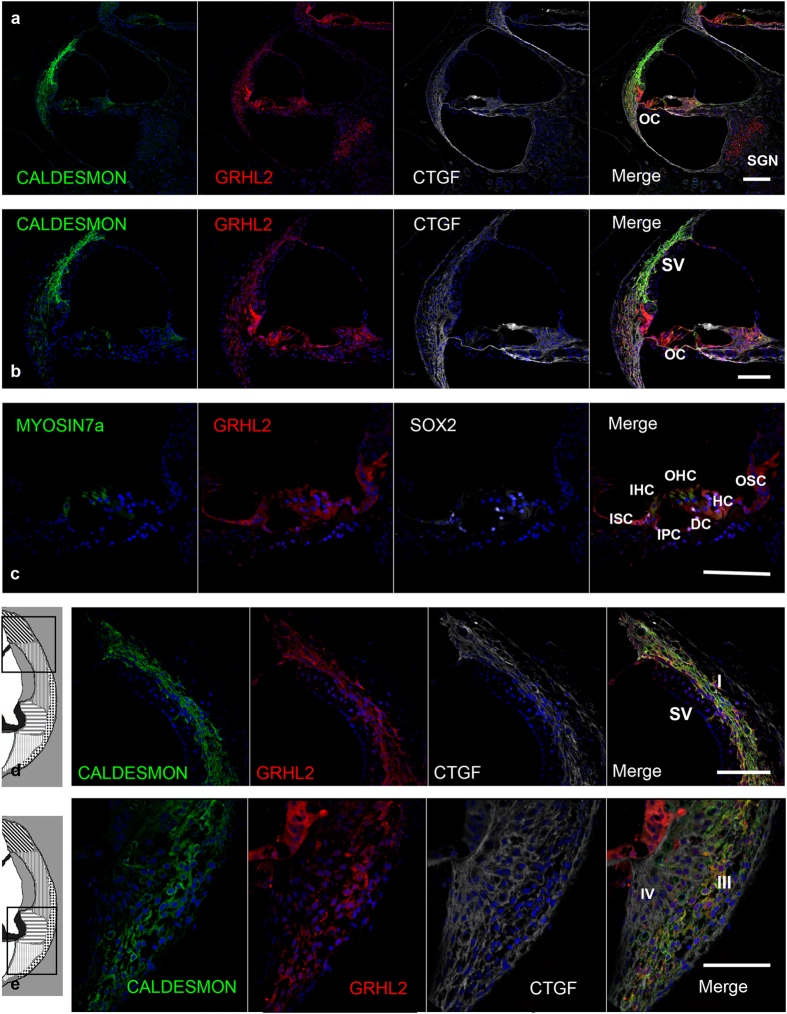
GRHL2 expression in the cochlea of the common marmoset (**a, b**) GRHL2 expression was observed in the lateral wall spiral ligament, hair cells, supporting cells, and spiral ganglion neurons. No expression was observed in the stria vascularis. (**c**) Supporting cell expression was broadly observed between the inner sulcus and outer sulcus cells, and GRHL2 expression was observed in the inner and outer hair cells. (**d,e**) In the spiral ligament, GRHL2 expression was observed in type I and III fibrocytes and co-localized with CALDESMON. The nuclei were counterstained with Hoechst (blue). Scale bar: 200 μm in a, 100 μm in (**b–e**). Middle turns in (**a–e**). SV: stria vascularis, OC: organ of Corti, SGN: spiral ganglion neurons, ISC: Inner sulcus cells, OSC: Outer sulcus cells, HC: Hensen’s cells, DC: Deiters’ cells, IPC: inner pillar cells, OHC: Outer hair cells, IHC: Inner hair cells, SLF: spiral ligament fibrocytes (I-V: Type I-V).

GRHL2, also known as TFCP2L3, is a transcriptional member of the Grh/CP2 family and is a mammalian homolog of the *Drosophila* gene *grainyhead*. In the common marmoset, GRHL2 expression was observed in the lateral wall spiral ligament, hair cells, supporting cells, and SGNs (Fig. 4a,b). No expression was observed in the stria vascularis, whereas it is expressed in the mouse stria vascularis. Supporting cell expression was broad, with both inner sulcus cells and outer sulcus cells expressing it, as well as inner and outer hair cells (Fig. 4c). In the spiral ligament, GRHL2 expression was observed in type I and III fibrocytes, which were co-labeled with CALDESMON (Fig. 4d,e). Notably, as shown in a previous report about human keratinocytes^26^, cytoplasmic expressions of GRHL2 were observed and we confirmed that this expression is not nonspecific signal using two different anti-GRHL2 antibodies (Supplementary Fig. 2).

### Expression pattern of the deafness gene DFNA5

**Figure 5:**
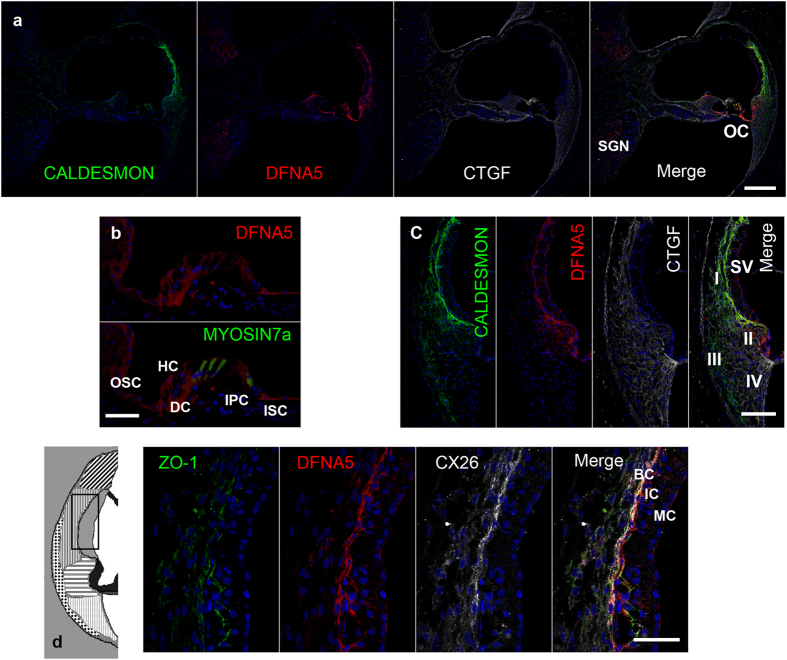
DFNA5 expression in the cochlea of the common marmoset (**a**) DFNA5 expression was observed in the spiral ligament fibrocytes, supporting cells, inner and outer hair cells, and spiral ganglion neurons. (**b**) Expression in the supporting cells was broadly observed between the inner sulcus and outer sulcus cells. (**c**) DFNA5 expression was observed in type II fibrocytes. (**d**) DFNA5 expression was observed in the ZO-1- and CX26-positive basal cells of the stria vascularis. The nuclei were counterstained with Hoechst (blue). Scale bar: 200 μm in (**a**), 100 μm in (**c**), 50 μm in (**b,d**). Middle turns in (**a,c**), apical turn in (**b**), and basal turn in d. SV: stria vascularis, OC: organ of Corti, SGN: spiral ganglion neurons, ISC: Inner sulcus cells, OSC: Outer sulcus cells, HC: Hensen’s cells, DC: Deiters’ cells, IPC: inner pillar cells, MC: Marginal cells, IC: Intermediate cells BC: basal cells, I-V: spiral ligament fibrocytes Type I-V.

*DFNA5* is the fifth mapped autosomal dominant locus for hereditary hearing impairment in humans^27^, yet its physiological function is still unknown. Its expression in mouse cochlea is thought to be in the stria vascularis^28^, but no detailed evaluation using immunostaining has ever been reported. In the common marmoset, we observed it in the stria vascularis, spiral ligament fibrocytes, both inner and outer hair cells, supporting cells, and SGNs (Fig. 5a). Expression was found in all types of the supporting cells between the inner sulcus and outer sulcus cell (Fig. 5b). In spiral ligaments, DFNA5 expression was limited to type II fibrocytes (Fig. 5c). In the marmoset stria vascularis, DFNA5 expression was limited to basal cells (Fig. 5d).

### Expression pattern of the deafness gene ATP6B1 (dRTA with Hearing loss)

**Figure 6:**
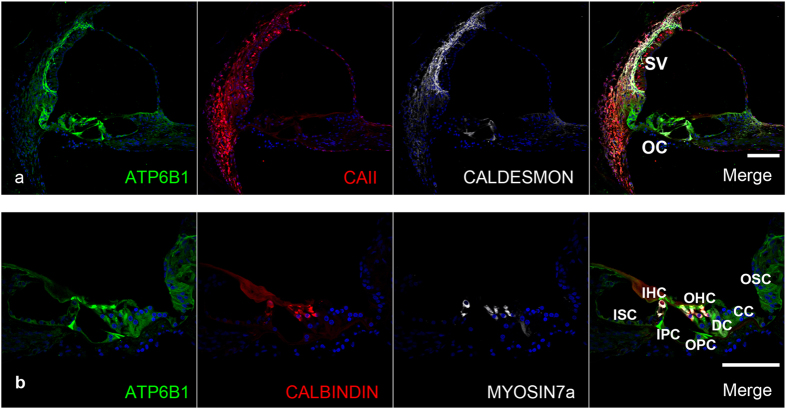
ATP6B1 expression in the cochlea of the common marmoset (**a**) ATP6B1 expression was observed in all types of spiral ligament fibrocytes, inner and outer hair cells, supporting cells, and the spiral limbus. (**b**) In the organ of Corti, ATP6B1 expression was observed in all hair cells and supporting cells between the inner sulcus and outer sulcus cells. The nuclei were counterstained with Hoechst (blue). Scale bar: 100 μm. Middle turn in (**a**) basal turn in (**b**). SV: stria vascularis, OC: organ of Corti, ISC: Inner sulcus cells, OSC: Outer sulcus cells, CC: Claudius cells, HC: Hensen’s cells, DC: Deiters’ cells, IPC: inner pillar cells, OHC: Outer hair cells, IHC: Inner hair cells.

ATP6B1 is one of the components of the vacuolar ATPase (V-ATPase), a multi-subunit enzyme that mediates acidification of eukaryotic intracellular organelles. In contrast to the limited expression in the spiral limbus in mouse^29^, expression in the marmoset was broadly observed in the lateral wall spiral ligament, in both inner and outer hair cells, in supporting cells, in the spiral limbus, and in SGNs (Fig. 6a). ATP6B1 expression in supporting cells was detected between the inner sulcus and outer sulcus cells (Fig. 6b).

### Expression pattern of the deafness gene COCH (DFNA9)

**Figure 7:**
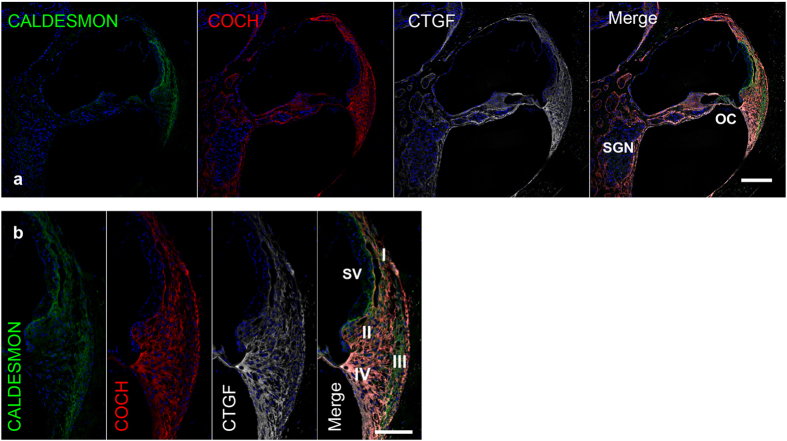
COCH expression in the cochlea of the common marmoset (**a,b**) COCH expression was observed in the spiral ligaments and spiral limbus. No expression was observed in epithelial cells including supporting cells and hair cells. The nuclei were counterstained with Hoechst (blue). Scale bar: 200 μm in (**a**) 100 μm in (**b**). Basal turn in (**a**) middle turn in (**b**). SV: stria vascularis, OC: organ of Corti, SGN: spiral ganglion neurons, I-V: spiral ligament fibrocytes Type I-V.

COCH (cochlin, coagulation factor C homology) is coded by the *COCH* gene. In humans, the expression of COCH is restricted to the inner ear^24^, and it is clinically used as a perilymph-specific protein^30^. Among the deafness genes tested in this experiment, this is the only gene for which a mouse model recapitulates the human symptoms^31^. In our study, the expression of COCH was observed in the spiral ligaments and spiral limbus (Fig. 7a,b). No expression was observed in epithelial cells. There was no difference between our results in the common marmoset and those reported for humans and mice^32^. The expression pattern of COCH appears to be conserved between rodents and non-human primates.

### Validations of antibodies used in this study

To validate specificities of the antibodies used in this study, we performed immunostaining of the skin, the small intestine or the kidney of common marmoset (Supplementary Fig. S3). Antibodies for CX31, CRYM and GRHL2 used in this study showed immunoreactivities in epidermis or hair follicles in the skin samples and no expression was detected in connective tissues as reported previously^33^,^34^,^35^,^36^. In small intestine samples, antibodies for DFNA5 used in this study showed immunoreactivities in villus and no immunoreactivities was detected in crypt as previously reported^37^.In kidney samples, antibodies for ATP6B1 used in this study showed immunoreactivities in collecting ducts and tubules and no immunoreactivities was detected in glomeruli as previously reported^38^. Additionally we performed antibody absorption tests with the antibodies which antigens used for producing them were commercially available. Immunoreactivities were abolished by incubation with antigen peptides (Supplementary Fig. S4). We also confirmed specificities of secondary antibodies used in this study (Supplementary Fig. S5).

## Discussion

Hearing loss is the most frequent congenital sensory impairment. Approximately 1 out of every 1000 newborns suffers from deafness^39^, and in about half the cases, the cause is monogenic^40^. Slowly progressive hereditary hearing loss may be accounted by the gradual loss of auditory cells thus treatments for preventing damage to the cells would be an effective therapeutic strategy. Here we examined the expression of 20 genes in the cochlea of the common marmoset and compared their patterns in between rodent and non-human primate cochleae by immunohistochemistry.

We chose six genes that cause congenital hereditary progressive hearing loss and we characterized their differential expression pattern between the common marmoset and rodents.

Mutations in the *GJB3* gene, which encodes CX31, cause DFNA2B/DFNB91. Hearing loss in DFNA2B has a progressive and down-sloping pattern. Remarkably, CX31 knockout mice do not suffer from hearing loss^5^; therefore, the detailed pathophysiology still remains to be elucidated, although disruptions in potassium recycling are suspected to be involved due to the function of CX31^41^. We found species-specific expression pattern of CX31 in cochleae. In mice, it is expressed not in the stria vascularis or the organ of Corti^23^. In the common marmoset, it is expressed in the basal cells of the stria vascularis and broadly in the supporting cells of the organ of Corti. These results suggest that the expression of CX31 in the organ of Corti plays a more essential role in the hearing of primates. The divergence of the connexin families during evolution, in their expression and functions, has been reported in the skin^33^,^42^ and heart^43^. Our findings suggest that the species-difference in the recycling of ions, due to the differential expression of these gap junction genes in the cochlea, accounts for the phenotypic difference between attempted mouse models and human cases – a conclusion that may bring us closer to new therapeutic strategies for preventing hearing loss.

Mutations in the *CRYM* gene cause progressive and down-sloping hearing loss^24^. However, knockout mouse models do not suffer from hearing loss^7^. In rodents, it is not expressed in the organ of Corti^24^,^25^. However, the common marmoset expressed it in the organ of Corti. A previous functional study found that CRYM binds thyroid hormone (T3), and suggested that *CRYM* mutations cause hearing loss through disturbing the thyroid hormone-binding properties of the cochlea^44^. Our results suggest that, in primates, the expression of CRYM and the subsequent interaction with thyroid hormone in the organ of Corti is essential for maintaining cochlear cells and preserving normal hearing.

Mutations in the *GRHL2* gene cause DFNA28, resulting in progressive, down-sloping hearing loss^45^. Knockout mouse model of *GRHL2* is embryonic lethal, and no established hearing loss model is available^46^. GRHL2 is a transcription factor that binds to and regulates the activity of the human telomerase reverse transcriptase gene promoter^47^ and age-related hearing loss genes^48^. The progressive hearing loss of DFNA28 and age-related hearing loss suggest that GRHL2 is essential for maintaining epithelial cells^49^. In mice, it is expressed in all epithelial cells of the cochlea^45^. In the common marmoset, GRHL2 was not expressed as broadly, and no expression was detected in the stria vascularis. Instead, it was expressed in the spiral ligament and limbus. Our detailed investigation into the expression patterns of GRHL2 will be useful for researching the disease mechanisms of DFNA28 and of age-related hearing loss, with our primate model offering more reliable information than rodents.

Mutations in *DFNA5* cause progressive, down-sloping hearing impairment^28^. *In vitro* studies have shown that the transfection of mutant *DFNA5* causes cell death, whereas transfection of wild-type *DFNA5* does not^50^. In addition, mice lacking *DFNA5* do not suffer from deafness^6^. Therefore, the mutations leading to *DFNA5*-induced hearing loss are thought to be gain-of-function mutations. In mice, it is expressed (at postnatal day 3) in the stria vascularis and the greater epithelial ridge, as determined by RT-PCR^27^, but the exact spatial expression pattern has not been characterized. We found that its expression in the stria vascularis was limited to the basal cells and it was also expressed in the SGNs. Considering the reported functional assay, knockout mouse model is not suitable and a gain-of-function mouse model with missense mutations will be awaited. Our detailed characterization of the expression pattern of DFNA5 will be useful for researching its role in disease.

Mutation of the *ATP6B1* gene causes dRTA with hearing loss. In autosomal recessive dRTA, a substantial fraction of the patients have sensorineural hearing loss with a progressive, down-sloping pattern^29^. Knockout mice model do not suffer from hearing loss^51^. In mice, it is expressed only in the spiral limbus^29^. In the common marmoset, it was detected not only in the spiral limbus, but also broadly across supporting cells.

Mutations in the *COCH* gene cause DFNA9, which is associated with progressive hearing loss^52^. Whereas knockout mice do not suffer from hearing loss^53^, mice carrying the same missense mutation as human patients show late onset hearing impairment^54^. We found no difference in the expression of the *COCH* gene between mouse, human^32^, and marmoset. This observation may account for rodent models being able to recapitulate human deafness; in other words, conservation of the expression pattern between primates and rodents should be a prerequisite for studying mouse models.

In this study, we analyzed six proteins causing progressive hereditary hearing loss, five of which (CX31, CRYM, GRHL2, DFNA5, and ATP6B1) do not recapitulate human symptoms in the mouse model (Table 1). Their amino acid sequences are much more similar to common marmoset than that of mouse (Supplementary Fig. S6) and immunostaining showed that the five genes had different expression patterns between the marmoset and mouse. On the other hand, the expression pattern of COCH, which has a mouse model that recapitulates the deafness phenotype of patients, was conserved between mouse, human, and marmoset (Supplementary Fig. S7).

Mouse mutants are a useful and powerful tool for examining the pathophysiologies of hereditary diseases, including deafness, but they are not always applicable. For missense mutations, a gain-of-function (including a dominant negative function) model may account for the symptoms, and, in these instances, a transgenic or knock-in approach is effective. For example, transgenic mice expressing a deafness-causing mutant of connexin 26, a mutant carrying the R75W mutation, have hearing impairments similar to those observed in human patients^55^. For nonsense or truncating mutations, a loss-of-function (*i.e*., knockout) model may sometimes explain the symptoms. For example, deficiency of Connexin 30 in mice causes severe hearing impairment as observed in human patients of *GJB6*, which is caused by mutations in *CX30*^56^. Choosing appropriate approaches regarding type of mutations in the human cases may offer rodent disease models.

However, these models are based on the assumption that the distributions of targeted gene products are same in humans and rodents, and we have found examples showing that this assumption is not always true by examining non-human primate cochleae. Considering the correlation of phenotypical difference and differential expression patterns, it may be helpful to examine the expression profiles of target genes in primates and rodents before creating a mouse mutant. The common marmoset model offers a useful platform to evaluate expression patterns in the primate because it avoids the difficulties of immunostaining human temporal bone, which is time-consuming and technically difficult.

Also, the difference in the life span of human and mouse may result in the failure of recapitulating human disease phenotypes in mice, especially for the bilateral progressive hearing loss or adult-onset hearing loss. In those diseases, the life-span of mouse may not be long enough considering the onset of the phenotypes. For these diseases, non-human primates, which have longer life span than mice, would be more appropriate. Another possibility that should be raised in the discussion of the discrepancies in phenotypes of human cases and mouse models is that of a human deafness gene being erroneously assigned as a deafness gene. Whether a mutation found in patients is pathogenic is essentially defined through an inductive process; thus, supporting experiments in disease models evaluating such mutation are of great importance. In this process, confirmations using human disease cells or non-human primate *in vivo* model will be helpful in the future. Knowledge of the inner ear has so far been largely dependent on experiments performed in rodent models (including mouse, rat, guinea pig, and gerbil), primarily because of well-established techniques in the biotechnology: The reagents are optimized to those species and the genetic modification is enabled. However, our results indicate that the difference in the expression patterns may lead to the phenotypical discrepancy between rodent models and human patients. We propose the common marmoset, a non-human primate that is easy to handle, as a promising option for bridging the species gap. Studies using genetically modified marmosets^57^,^58^ would be a fascinating and feasible strategy for elucidating the mechanisms of hereditary hearing loss that cannot be determined by using rodent models.

## Methods

### Specimens of the common marmoset

Fixed and decapitated cadaverous heads of the 3–6 years old common marmosets were kindly provided from Reona Kobayashi, Takahiro Kondo, Kimika Yoshino-Saito, and Seiji Shiozawa. Fixed small intestine and kidney samples were also kindly provided from them. The animal experiments were approved by the ethics committee of Keio University (number: 11006) and were in accordance with the guidelines of the National Institutes of Health, and the Ministry of Education, Culture, Sports, Science and Technology of Japan.

### Tissue preparation

Temporal bones from young adult marmosets were dissected, fixed, decalcified with Decalcifying Solution B (Wako, Saitama, Japan) for 3–4 weeks, and embedded in Tissue-Tek O.C.T. compound (Sakura Finetek, Tokyo, Japan) for cryosection. The 7 μm sections were used for immunohistochemistry.

Small intestines, kidneys and facial skins from young adult marmosets were used for validations of antibodies. Those tissues were dissected and fixed with 4% PFA, and the specimens were embedded in Tissue-Tek O.C.T. compound for cryosection. The 7 μm sections were used for immunohistochemistry.

Temporal bones from 3 weeks old mice (C57BL/6) were dissected, fixed, decalcified with Decalcifying Solution B for 3 days, and embedded in Tissue-Tek O.C.T. compound for cryosection. The 7 μm sections were used for immunohistochemistry. This animal experiments using mice were approved by the ethics committee of Keio University (number: 08020) and were in accordance with the guidelines of the National Institutes of Health, and the Ministry of Education, Culture, Sports, Science and Technology of Japan.

### Immunohistochemistry

After a brief wash with PBS, sections were heated (80 °C) in 10 mM citrate buffer (pH 6) for 1 h. After a brief wash, the sections were preblocked for 1 h at room temperature with 10% normal serum in PBS, incubated with primary antibodies at 4 °C overnight, and incubated with Alexa Fluor-conjugated secondary antibodies (Alexa488, Alexa555 and Alexa647) for 60 min at room temperature. The nuclei were counterstained with Hoechst 33342. For validations of secondary antibodies immunohistochemistry was performed without primary antibody in an overnight incubation step.

### Antibody absorption test

Primary antibodies were incubated with or without (control) peptide used for generating antigens provided from antibody suppliers. After overnight incubations following immunohistochemical procedures were performed with using these antibody as primary antibody.

### Antibodies

The primary antibodies used in this study are as follows: anti-SOX2 (goat IgG, Santa Cruz Biotechnology, Dallas, TX, USA, sc17320, 1:100), anti-CX26 (mouse IgG, Zymed, San Francisco, CA, USA, 13–8100, 1:300), anti-CX30 (rabbit IgG, Sigma-Aldrich, St. Louis, MO, USA, HPA014846, 1:200), anti-CX31 (rabbit IgG, Proteintech, Manchester, UK, 12880–1-AP, 1:100), anti-CTGF (goat IgG, Santa Cruz Biotechnology, sc14939, 1:100), anti-AQP1 (rabbit IgG, Millipore, Billerica, MA, USA, AB3272, 1:300), anti-CALDESMON (Sigma-Aldrich, C0297, 1:100), anti-CA II (rabbit IgG, Santa Cruz Biotechnology, sc25596, 1:100), anti-NKCC1 (goat IgG, Santa Cruz Biotechnology, sc21545, 1:300), anti-Na+/K+-ATPase α1 (rabbit IgG, Novus Biologicals, Littleton, CO, USA, EP1845Y, 1:500), anti-CALBINDIN (rabbit IgG, Abcam, Cambridge, UK, ab11426, 1:1000), anti-ZO-1 (goat IgG, Abcam, ab190085, 1:100), anti-CRYM (mouse IgG, GeneTex, Irvine, CA, USA, GTX84654, 1:100), anti-MYOSIN 7a (mouse IgG, DSHB, Iowa City, IA,USA, 138–1-s, 1:30; rabbit IgG, Proteus, 25–6790), anti-GRHL2 (rabbit IgG, Sigma-Aldrich, HPA004820, 1:200, rabbit IgG, LSBio, LS-B3983, 1:150), anti-DFNA5 (rabbit IgG, Sigma-Aldrich, HPA011326, 1:400), anti-COCH (rabbit IgG, Sigma-Aldrich, HPA050122, 1:100), anti-β-TUBULIN III (mouse IgG, Sigma-Aldrich, T8660, 1:250), anti-ATP6B1 (goat IgG, Santa Cruz Biotechnology, sc21206, 1:50), and anti-PERIPHERIN (rabbit IgG, Millipore, AB1530, 1:100).

### Antigen peptides

For GRHL2, PrEST Antigen GRHL2 (Sigma-Aldrich, APREST86696) and for ATP6B1 (Santa Cruz Biotechnology, sc21206P).

## Additional Information

**How to cite this article**: Hosoya, M. *et al.* Distinct Expression Patterns Of Causative Genes Responsible For Hereditary Progressive Hearing Loss In Non-Human Primate Cochlea. *Sci. Rep.* **6**, 22250; doi: 10.1038/srep22250 (2016).

## Supplementary Material

Supplementary Information

## Figures and Tables

**Table 1:**
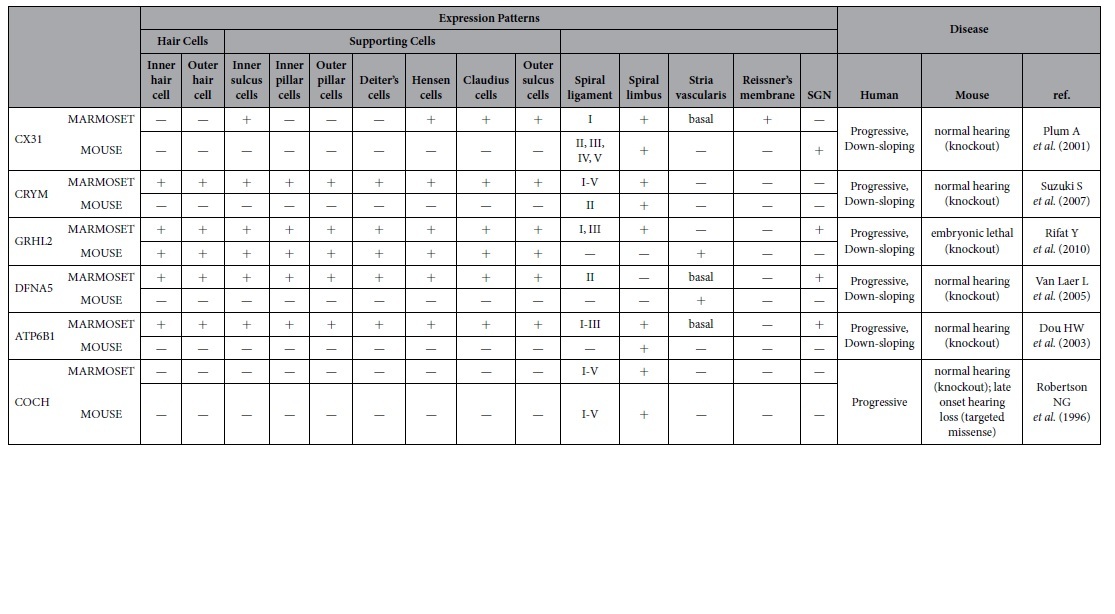
Differential expression of deafness genes in marmosets and rodents.
