# Supplementary material for: Distinct Expression Patterns Of Causative Genes Responsible For Hereditary Progressive Hearing Loss In Non-Human Primate Cochlea
Source: Sci Rep. 2016 Feb 26;6:22250. doi: 10.1038/srep22250 (PMC4768099; doi:10.1038/srep22250)
Supplement: Supplementary Information [file srep22250-s1.pdf]

Distinct Expression Patterns Of Causative Genes Responsible For Hereditary Progressive Hearing Loss In Non-Human Primate Cochlea

Makoto Hosoya[1], Masato Fujioka[1], Kaoru Ogawa[1], and Hideyuki Okano[2,*]

1 Keio University School of Medicine, Department of Otorhinolaryngology, Head and Neck Surgery, 35 Shinanomachi Shinjyuku-ku Tokyo, 160-8582, Japan

2 Keio University School of Medicine, Department of Physiology, 35 Shinanomachi Shinjyuku-ku Tokyo, 160-8582, Japan

*hidokano@a2.keio.jp

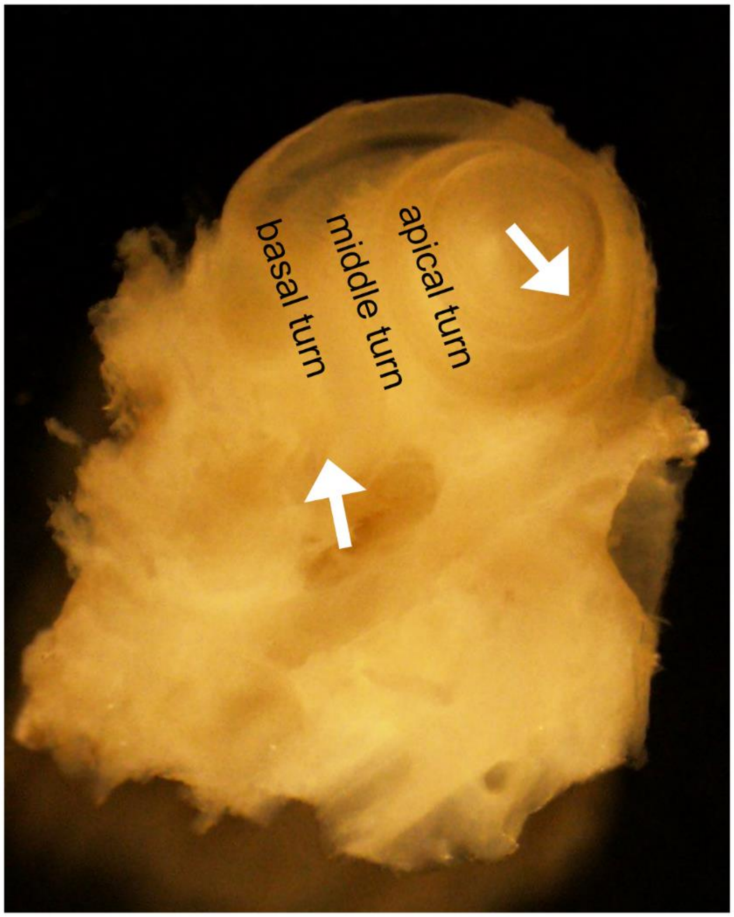

Supplementary Figure S.1

The common marmoset cochlea

The common marmoset cochlea has 2 and 3/4 turns (between allows) with apical, middle and basal turns as similar to that of human. Note that the cochlear bone capsule was removed.

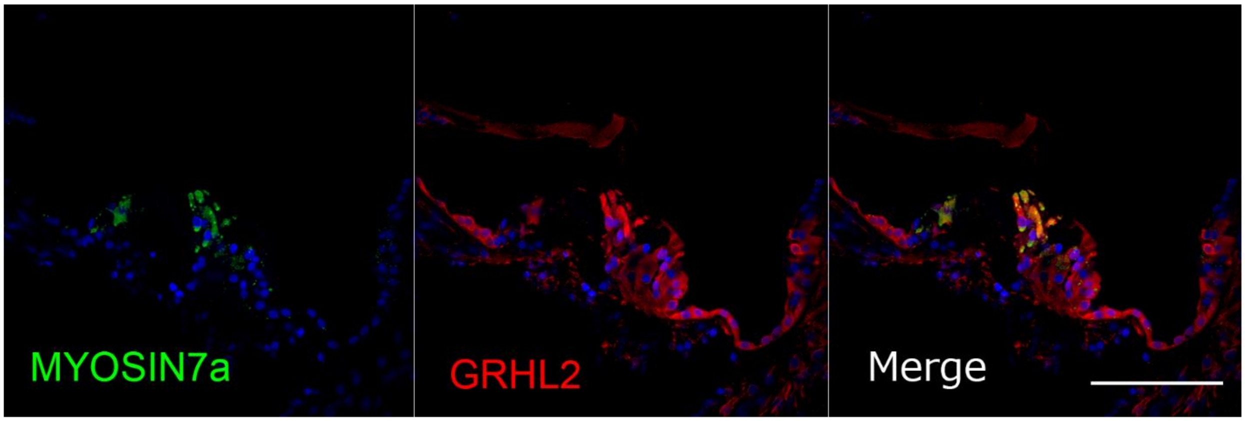

Supplementary Figure. S2

Cytoplasmic expression of GRHL2

Immunostaining for GRHL2 was observed in the nuclear and cytoplasm. We confirmed the localization by using two different antibodies. Like as shown in Fig.4 (using antibody from Sigma-Aldrich), the immunoreactivity was also detected in cytoplasm with another anti GRHL2 antibody from LSBio. The nuclei were counterstained with Hoechst (blue). Scale bar: 100 μm.

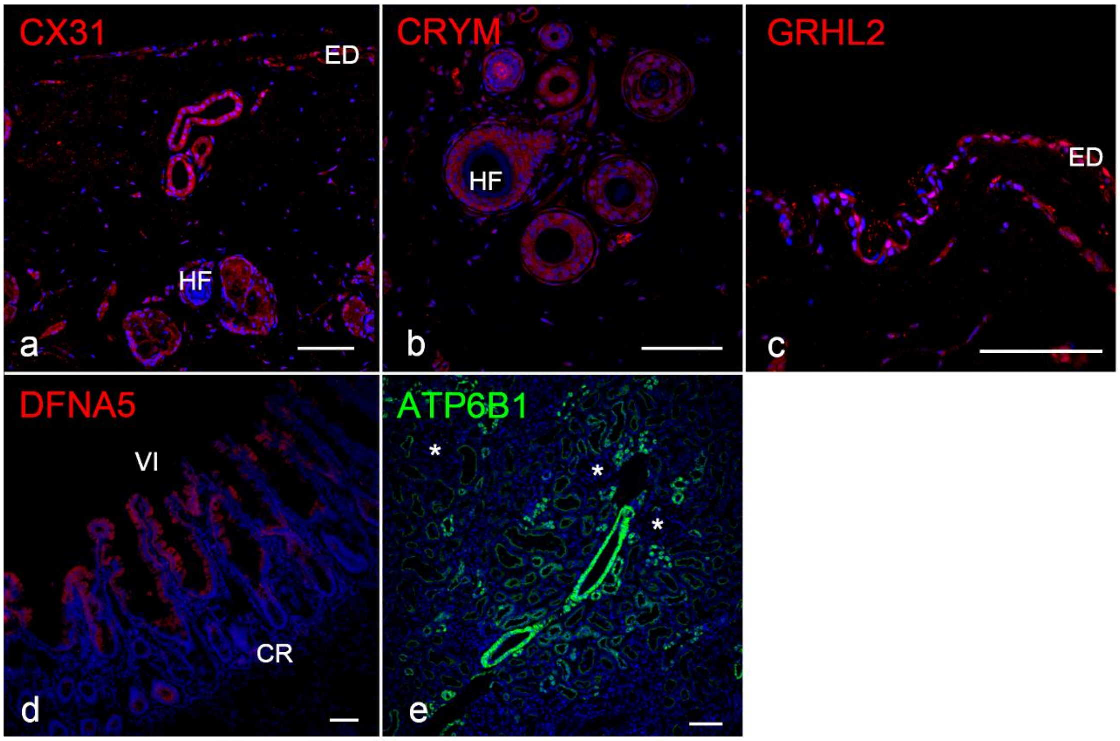

Supplementary Figure. S3

Expressions of CX31, CRYM and GRHL2 in the skin, DFNA5 in the small intestine and ATP6B1 in the kidney

For validating antibodies specificities, we performed immunostaining of the skin, the small intestine and the kidney of common marmoset. (a-c) CX31, CRYM and GRHL2 immunoreactivities were detected in hair follicles (HF) or epidermal layers (ED) and not detected in surrounding connective tissues. (d) DFNA5 immunoreactivities were detected in villus (VI) and not detected in crypt (CR) of the small intestine. (e) ATP6B1 immunoreactivities were detected in collecting ducts and tubules and no immunoreactivities was detected in glomeruli (*). The nuclei were counterstained with Hoechst (blue). Scale bar: 100 μm.

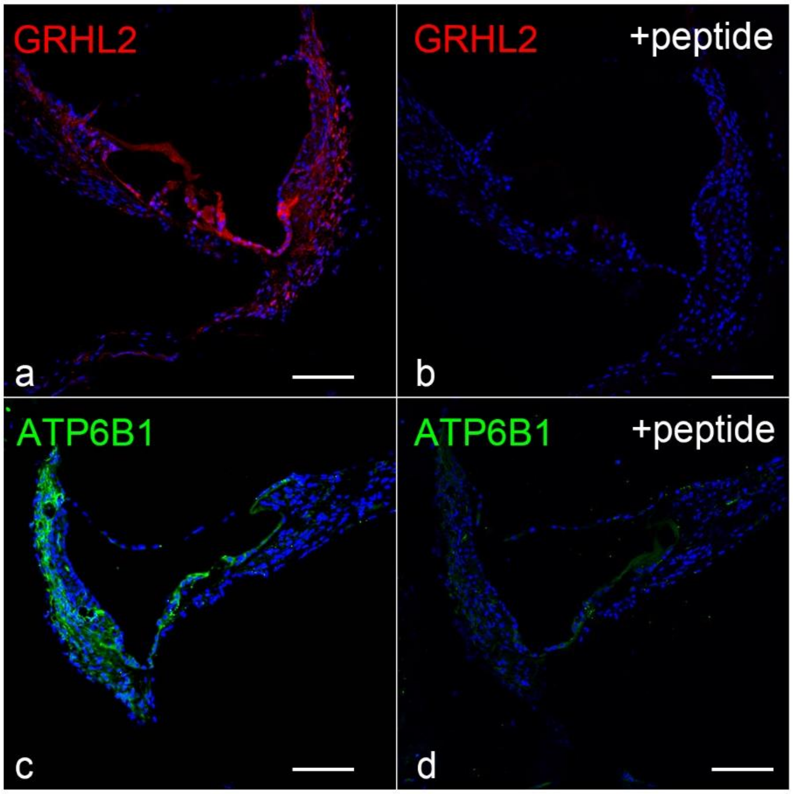

Supplementary Figure. S4

Antibody absorption test by antigen peptide

For validating antibodies specificities, we performed antibody absorption test by antigen peptide. Immunoreactivities (a and c) were abolished by incubations with antigen peptide (b and d). The nuclei were counterstained with Hoechst (blue). Scale bar: 100 µm.

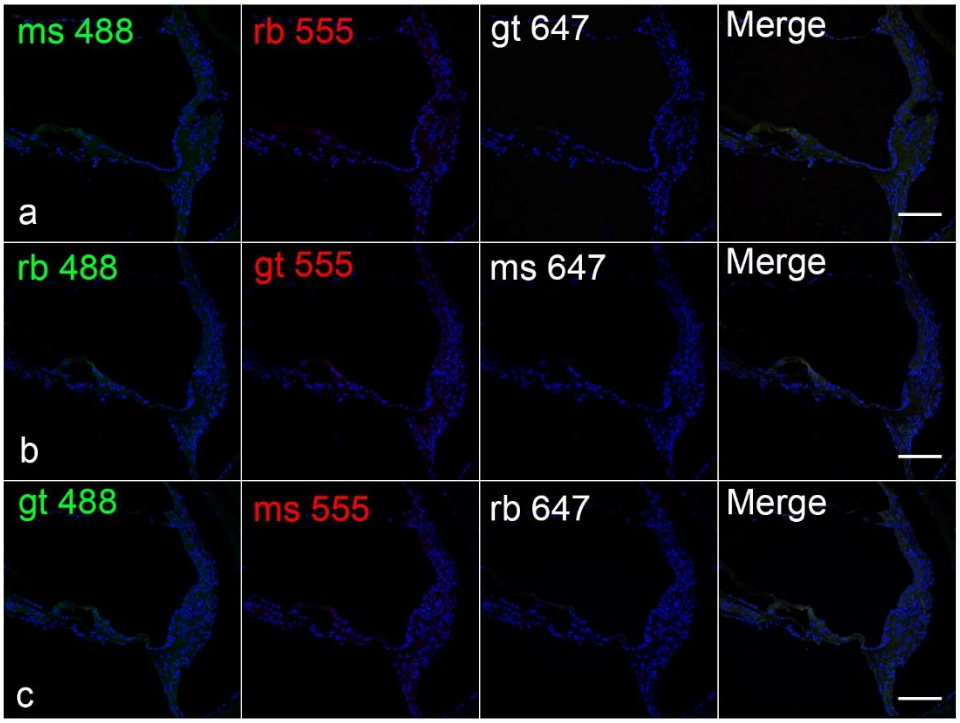

Supplementary Figure. S5

Validations of secondary antibodies.

For validating secondary antibodies' specificities, we performed immunostaining without primary antibody incubations. No immunoreactivities were observed. The nuclei were counterstained with Hoechst (blue). Scale bar: 100 µm. ms: anti-mouse IgG rb: anti-rabbit Ig G gt: anti-goat IgG, 488: Alexa 488, 555: Alexa 555, 647: Alexa 647

Supplementary Figure. S6

a. CX31

Homo sapiens	MDWKTLQALLSGVNKYSTAFGRIWLSVVFVFWVLVYVVAAERVWGDEQKD	50
Callithrix jacchus	MDWKTLQALLSGVNKYSTAFGRIWLSVVFVFRVLVYVVAAERVWGDEQKD	50
Mus musculus	MDWKKLQDLLSGVNQYSTAFGRIWLSVVFVFRVLVYVVAAERVWGDEQKD	50
Homo sapiens	FDCNTKQPGCTNVCYDNYFPISNIRLWALQLIFVTCPSLLVILHVAYREE	100
Callithrix jacchus	FDCNTKQPGCTNVCYDNFFPISNIRLWALQLIFVTCPSLLVILHVAYREE	100
Mus musculus	FDCNTRQPGCTNVCYDNFFPISNIRLWALQLIFVTCPSMLVILHVAYREE	100
Homo sapiens	RERRHRQKHGDQCAKLYDNAGKKHGGLWWTYLFSLIFKLIIEFLFLYLLH	150
Callithrix jacchus	RERRHRQKHGDQCSKLYDNAGKKHGGLWWTYLFSLIFKLIIEFLFLYLLH	150
Mus musculus	RERKHRQKHGEQCAKLYSHPGKKHGGLWWTYLFSLIFKLIIELVFLYVLH	150
Homo sapiens	TLWHGFNMPRLVQCANVAPCPNIVDCYIARPTEKKIFTYFMVGASAVCIV	200
Callithrix jacchus	TLWHGFDMPRLVQCANVAPCPNIVDCYIARPTEKKIFTYFMVGASAVCIV	200
Mus musculus	TLWHGFTMPRLVQCASIVPCPNTVDCYIARPTEKKVFTYFMVGASAVCII	200
Homo sapiens	LTICELCYLICHRVLRGLHKDKPRGGCSPSSSASRASTCRCHHKLVEAGE	250
Callithrix jacchus	LTICELCYLICHRVLRALRKDKPQGGHTPSSFASRASTCRCHHKLVEAGE	250
Mus musculus	LTICEICYLIFHRIMRGISKGKSTKSISSPKSSSRASTCRCHHKLLESGD	250
Homo sapiens	VDPDPGNNKLQASAPNLTPI	270
Callithrix jacchus	LDPHPGNNKLQASAPNMTPI	270
Mus musculus	PEADPASEKLQASAPSLTPI	270

b. CRYM

Homo sapiens	MSRVPAFLSAAEVEEHLRSSSLLIPPLETALANFSSGPEGGVMQPVRTVV	50
Callithrix jacchus	MSRVPAFLSAAEVEEHLRSSSLLIPPLETALANFSSGPEGGVMQPVRTVV	50
Mus musculus	MKRAPAFLSAEEVQDHLRSSSLLIPPLEAALANFSKGPDGGVMQPVRTVV	50
Homo sapiens	PVTKHRGYLGVMPAYSAAEDALTTKLVTFYEDRGITSVVPSHQATVLLFE	100
Callithrix jacchus	PVTKHRGYLGVMPAYSAAEDALTTKLVTFYEDRGITSIVPSHQATVLLFE	100
Mus musculus	PVAKHRGFLGVMPAYSAAEDALTTKLVTFYEGHSNT-AVPSHQASVLLFD	99
Homo sapiens	PSNGTLLAVMDGNVITAKRTAAVSAIATKFLKPPSSEVLCILGAGVQAYS	150
Callithrix jacchus	PSNGTLLAVMDGSVITAKRTAAVSAIATKFLKPPSSEVLCILGAGVQAYS	150
Mus musculus	PSNGSLLAVMDGNVITAKRTAAVSAIATKLLKPPGSDVLCILGAGVQAYS	149
Homo sapiens	HYEIFTEQFSFKEVRIWNRTKENAEKFADTVQGEVRVCSSVQEAVAGADV	200
Callithrix jacchus	HYEIFTEQFSFKEVRIWNRTKENAEKFADTVQGEVRVCSSVQEAVTGADV	200
Mus musculus	HYEIFTEQFSFKEVRMWNRTRENAEKFASTVQGDVRVCSSVQEAVTGADV	199
Homo sapiens	IITVTLATEPILFGEWVKPGAHINAVGASRPDWRELDDELMKEAVLYVDS	250
Callithrix jacchus	IITVTLATEPILFGEWVKPGAHINAVGASRPDWRELDDELMKEAVLYVDS	250
Mus musculus	IITVTMATEPILFGEWVKPGAHINAVGASRPDWRELDDELMRQAVLYVDS	249
Homo sapiens	QEAALKESGDVLLSGAEIFAELGEVIKGVKPAHCEKTTVFKSLGMAVEDT	300
Callithrix jacchus	QEAALKESGDVLLSGAEIFAELGEVIKGVKPAHCEKTTVFKSLGMAVEDT	300
Mus musculus	REAALKESGDVLLSGADIFAELGEVISGAKPAHCEKTTVFKSLGMAVEDL	299
Homo sapiens	VAAKLIYDSWSSGK	314
Callithrix jacchus	VAAKLIYDSWSSGK	314
Mus musculus	VAAKLVYDSWSSGK	313

c. GRHL2

```
Homo sapiens         T L E A T K S L R Q K Q G E G P M T Y L N K G Q F Y A I T L S E T G D N K C F R H P I S K V R S V V   300
Callithrix jacchus   T L E A T K S L R Q K Q G E G P M T Y L N K G Q F Y A I T L S E T G D N K C F R H P I S K V R S V V   300
Mus musculus         T L E A T K S L R Q K Q G E G P M T Y L N K G Q F Y A I T L S E T G D N K C F R H P I S K V R S V V   300

Homo sapiens         M V V F S E D K N R D E Q L K Y W K Y W H S R Q H T A K Q R V L D I A D Y K E S F N T I G N I E E I   350
Callithrix jacchus   M V V F S E D K N R D E Q L K Y W K Y W H S R Q H T A K Q R V L D I A D Y K E S F N T I G N I E E I   350
Mus musculus         M V V F S E D K N R D E Q L K Y W K Y W H S R Q H T A K Q R V L D I A D Y K E S F N T I G N I E E I   350

Homo sapiens         A Y N A V S F T W D V N E E A K I F I T V N C L S T D F S S Q K G V K G L P L M I Q I D T Y S Y N N   400
Callithrix jacchus   A Y N A V S F T W D V N E E A K I F I T V N C L S T D F S S Q K G V K G L P L M I Q I D T Y S Y N N   400
Mus musculus         A Y N A V S F T W D V N E E A K I F I T V N C L S T D F S S Q K G V K G L P L M I Q I D T Y S Y N N   400

Homo sapiens         R S N K P I H R A Y C Q I K V F C D K G A E R K I R D E E R K Q N R K K G K G Q A S Q T Q C N S S S   450
Callithrix jacchus   R S N K P I H R A Y C Q I K V F C D K G A E R K I R D E E R K Q N R K K G K G Q T S Q T Q C N S S S   450
Mus musculus         R S N K P I H R A Y C Q I K V F C D K G A E R K I R D E E R K Q N R K K G K G Q A S Q A Q C N N S S   450

Homo sapiens         D G K L A A I P L Q K K S D I T Y F K T M P D L H S Q P V L F I P D V H F A N L Q R T G Q V Y Y N T   500
Callithrix jacchus   D G K L A A I P L Q K K S D I T Y F K T M P D L H S Q P V L F I P D V H F A N L Q R T G Q V Y Y N T   500
Mus musculus         D G K M A A I P L Q K K S D I T Y F K T M P D L H S Q P V L F I P D V H F A N L Q R T G Q V Y Y N T   500

Homo sapiens         D D E R E G G S V L V K R M F R P M E E E F G P V P S K Q M K E E G T K R V L L Y V R K E T D D V F   550
Callithrix jacchus   D D E R E G - S V L V K R M F R P M E E E F G P V P S K Q M K E E G T K R V L L Y V R K E T D D V F   549
Mus musculus         D D E R E G S S V L V K R M F R P M E E E F G P T P S K Q I K E E N V K R V L L Y V R K E N D D V F   550

Homo sapiens         D A L M L K S P T V K G L M E A I S E K Y G L P V E K I A K L Y K K S K K G I L V N M D D N I I E H   600
Callithrix jacchus   D A L M L K S P T V K G L M E A I S E K Y G L P V E K I A K L Y K K S K K G I L V N M D D N I I E H   599
Mus musculus         D A L M L K S P T V K G L M E A L S E K Y G L P V E K I T K L Y K K S K K G I L V N M D D N I I E H   600

Homo sapiens         Y S N E D T F I L N M E S M V E G F K V T L M E I   625
Callithrix jacchus   Y S N E D T F I L N M E S M V E G F K V T L M E I   624
Mus musculus         Y S N E D T F I L N M E S M V E G F K I T L M E I   625
```

d. DFNA5

```
Homo sapiens        MFAKATRNFLREVDADGDLIAVSNLNDSDKLQLLSLVTKKKRFWCWQRPK   50
Callithrix jacchus  MFAKATKNFLREVDADGDLIAVSNLNDSDKLQLLSLVTKKKRFWCWQRPK   50
Mus musculus        MFAKATRNFLKEVDAGGDLISVSHLNDSDKLQLLSLVTKKKRYWCWQRPK   50

Homo sapiens        YQFLSLTLGDVLIEDQFPSPVVVESDFVKYEGKFANHVSGTLETALGKVK   100
Callithrix jacchus  YQFLSITLGDVLKEDQFPSPVVVESDFVKYESKFANHVSGTLDTALGKVK   100
Mus musculus        YQILSATLEDVLTEGHCLSPVVVESDFVKYESKCENHKSGAIGTVVGKVK   100

Homo sapiens        LNLGGSSRVESQSSFGTLRKQEVDLQQLIRDSAERTINLRNPVLQQVLEG   150
Callithrix jacchus  LNVGGSSRVESQSSFGTLRKQEVDLQQLIRDSAERTINLRNPVLQQVLEE   150
Mus musculus        LNVGGKGVVESHSSFGTLRKQEVDVQQLIQDAVKRTVNMDNLVLQQVLES   150

Homo sapiens        RNEVLCVLTQKITTMQKCVISEHMQVEEKCGGIVGIQTKTVQVSATEDGN   200
Callithrix jacchus  RNEVLCVLTQKITTMQKCVISEHTQVEEKCGGMVGIQTKTVQVSATEDGN   200
Mus musculus        RNEVLCVLTQKIMTTQKCVISEHVQSEETCGGMVGIQTKTIQVSATEDGT   200

Homo sapiens        VTKDSNVVLEIPAATTIAYGVIELYVKLDGLFEFCLLRGKQGGFENKKRI   250
Callithrix jacchus  VMKDSNVVLEIPAATTIAYGVIELYVKLDGHFEFCLLQGKESGFENKRRI   250
Mus musculus        VTTDTNVVLEIPAATTIAYGIMELFVKQDGQFEFCLLQGKHGGFEHERKL   250

Homo sapiens        DSVYLDHLVFREFAFIDMPDAAHGISSQDGPLSVLKQATLLLERNFHPFA   300
Callithrix jacchus  DSVYLDPLAFREFAFIDMPDAGHGISSQDGPLSVLKQATPLLERNFHPFV   300
Mus musculus        DSVYLDPLAYREFAFLDMPDGGQGISSQDGPLRVVKQATLHLERSFHPFA   300

Homo sapiens        ELPEPQQTALSDIFQAVVFDDELLMVLEPVCDDLVSGLSPTVAVLG--EL   348
Callithrix jacchus  ELPEPQQTALSDIFQAVLFDNELLMVLEPVCDDVVSGLSPPLVVLG--EL   348
Mus musculus        VLPAQQQRALFCVLQKILFDEELLRALEQVCDDVAGGLWSSQAVLAMEEL   350

Homo sapiens        KPRQQQDLVAFLQLVGCSLQGGCPGPEDAGSKQ-LFMTAYFLVSALAEMP   397
Callithrix jacchus  MPRQQQDVMAFLQLVGCSLQGERPGPEDAGSKR-LFTTAYFLVSALAEMP   397
Mus musculus        TDSQQQDLTAFLQLVGYRIQGEHPGPQDEVSNQKLFATAYFLVSALAEMP   400

Homo sapiens        DSAAALLGTCCKLQIIPTLCHLLRALSDDGVSDLEDPTLTPLKDTERFGI   447
Callithrix jacchus  DNAAALLGTCYKLQIIPTLCHLLRALSDDGVSDLEDPTLAPLKDTERFGI   447
Mus musculus        DNATVFLGTCCKLHVISSLCCLLHALSDDSVCDFHNPTLAPLRDTERFGI   450

Homo sapiens        VQRLFASADISLERLKSSVKAVILKDSKVFPLLLCITLNGLCALGREHS-   496
Callithrix jacchus  VQRLFASADITLERLKSSVKAVILKDSEIFPLLLCITLNGLCALGREHS-   496
Mus musculus        VQRLFASADIALERMQFSAKATILKDSCIFPLILHITLSGLSTLSKEHEE   500

Homo sapiens        ------------
Callithrix jacchus  ------------
Mus musculus        ELCQSGHATGQD   512
```

e. ATP6B1

Homo sapiens	M A M E I D S R P G G L P G S S C N L G A A R E H M Q A V T R N Y I T H P R V T Y R T V C S V N G P	50
Callithrix jacchus	M A M E I D S R P G G L P G S G F K L G A A R E H M Q A V T R N Y I T H P R V T Y R T V C S V N G P	50
Mus musculus	M A T T V D S R S S G F T G N S C D P G T A Q E H V Q A V T R N Y I T H P R V T Y R T V C S V N G P	50
Homo sapiens	L V V L D R V K F A Q Y A E I V H F T L P D G T Q R S G Q V L E V A G T K A I V Q V F E G T S G I D	100
Callithrix jacchus	L V V L D Q V K F A Q Y A E I V H F T L P D G T Q R S G Q V L E V A G T K A V V Q V F E G T S G I D	100
Mus musculus	L V V L D Q V K F A Q Y A E I V N F T L P D G T Q R S G Q V L E V A G T K A I V Q V F E G T S G I D	100
Homo sapiens	A R K T T C E F T G D I L R T P V S E D M L G R V F N G S G K P I D K G P V V M A E D F L D I N G Q	150
Callithrix jacchus	A R K T T C E F T G D I L R T P V S E D M L G R V F N G S G K P I D K G P V V M A E D F L D I N G Q	150
Mus musculus	S Q K T T C E F T G D I L R T P V S E D M L G R I F N G S G K P I D K G P A V M A E E F L D I N G Q	150
Homo sapiens	P I N P H S R I Y P E E M I Q T G I S P I D V M N S I A R G Q K I P I F S A A G L P H N E I A A Q I	200
Callithrix jacchus	P I N P H S R I Y P E E M I Q T G I S P I D V M N S I A R G Q K I P I F S A A G L P H N E I A A Q I	200
Mus musculus	P I N P H D R I Y P E E M I Q T G I S P I D V M N S I A R G Q K I P I F S A A G L P H N E I A A Q I	200
Homo sapiens	C R Q A G L V K K S K A V L D Y H D D N F A I V F A A M G V N M E T A R F F K S D F E Q N G T M G N	250
Callithrix jacchus	C R Q A G L V K K S K A V L D Y H D D N F A I V F A A M G V N M E T A R F F K S D F E Q N G T M G N	250
Mus musculus	C R Q A G L V K K S K A V L D Y H E D N F A I V F A A M G V N M E T A R F F K S D F E Q N G T M G N	250
Homo sapiens	V C L F L N L A N D P T I E R I I T P R L A L T T A E F L A Y Q C E K H V L V I L T D M S S Y A E A	300
Callithrix jacchus	V C L F L N L A N D P T I E R I I T P R L A L T T A E F L A Y Q C E K H V L V I L T D M S S Y A E A	300
Mus musculus	V C L F L N L A N D P T I E R I I T P R L A L T T A E F L A Y Q C E K H V L V I L T D M S S Y A E A	300
Homo sapiens	L R E V S A A R E E V P G R R G F P G Y M Y T D L A T I Y E R A G R V E G R G G S I T Q I P I L T M	350
Callithrix jacchus	L R E V S A A R E E V P G R R G F P G Y M Y T D L A T I Y E R A G R V E G R G G S I T Q I P I L T M	350
Mus musculus	L R E V S A A R E E V P G R R G F P G Y M Y T D L A T I Y E R A G R V E G R G G S I T Q I P I L T M	350
Homo sapiens	P N D D I T H P I P D L T G F I T E G Q I Y V D R Q L H N R Q I Y P P I N V L P S L S R L M K S A I	400
Callithrix jacchus	P N D D I T H P I P D L T G F I T E G Q I Y V D R Q L H N R Q I Y P P I N V L P S L S R L M K S A I	400
Mus musculus	P N D D I T H P I P D L T G F I T E G Q I Y V D R Q L H N R Q V Y P P I N V L P S L S R L M K S A I	400
Homo sapiens	G E G M T R K D H G D V S N Q L Y A C Y A I G K D V Q A M K A V V G E E A L T S E D L L Y L E F L Q	450
Callithrix jacchus	G E G M T R K D H G D V S N Q L Y A C Y A I G K D V Q A M K A V V G E E A L T S E D L L Y L E F L Q	450
Mus musculus	G E G M T R K D H G D V S N Q L Y A C Y A I G K D V Q A M K A V V G E E A L T S E D L L Y L E F L Q	450
Homo sapiens	K F E K N F I N Q G P Y E N R S V F E S L D L G W K L L R I F P K E M L K R I P Q A V I D E F Y S R	500
Callithrix jacchus	K F E K N F I N Q G P Y E N R S V F E S L D L G W K L L R I F P K E M L K R I P Q A M I D E F Y S R	500
Mus musculus	K F E K N F I T Q G P Y E N R T V F E S L D L G W K L L R I F P K E M L K R I P Q S M T D E F Y S R	500
Homo sapiens	E G A L Q D L A P D T A L	513
Callithrix jacchus	E G A P Q D P A P D T A L	513
Mus musculus	Q G A Q Q D P A S D T A L	513

f. COCH

Homo sapiens	M S A A W I P A L G L G V C L L L L P G P - - A G S E G A A P I A I T C F T R G L D I R K E K A D V	48
Callithrix jacchus	M S A A W I Q T L F L G V C L Q L L P G P - - M G S E G A A P I A I T C F T R G L D I R K E K A D V	48
Mus musculus	M P S S R I P A L C L G A W L L L L L L P R F A R A E G A V P I P V T C F T R G L D I R K E K A D V	50
Homo sapiens	L C P G G C P L E E F S V Y G N I V Y A S V S S I C G A A V H R G V I S N S G G P V R V Y S L P G R	98
Callithrix jacchus	L C P G G C P L E E F S V Y G N I V Y A S V S S I C G A A V H R G V I S N S G G P V R V Y S L P G R	98
Mus musculus	L C P G G C S L E E F S V F G N I V Y A S V S S I C G A A V H R G V I G T S G G P V R V Y S L P G R	100
Homo sapiens	E N Y S S V D A N G I Q S Q M L S R W S A S F T V T K G K S S T Q E A T G Q A V S T A H P P T G K R	148
Callithrix jacchus	E N Y S S V D A N G I Q S Q M L S R W S A S F T V T K G K S S T Q E A T G Q A V S T A H P P T G K R	148
Mus musculus	E N Y S S V D A N G I Q S Q M L S R W S A S F A V T K G K S S T Q E A T G R A V S T A H P P S G K R	150
Homo sapiens	L K K T P E K K T G N K D C K A D I A F L I D G S F N I G Q R R F N L Q K N F V G K V A L M L G I G	198
Callithrix jacchus	L K K T P E K K T G N K D C K A D I A F L I D G S F N I G Q R R F N L Q K N F V G K V A L M L G I G	198
Mus musculus	L K K T P E K K T G N K D C K A D I A F L I D G S F N I G Q R R F N L Q K N F V G K V A L M L G I G	200
Homo sapiens	T E G P H V G L V Q A S E H P K I E F Y L K N F T S A K D V L F A I K E V G F R G G N S N T G K A L	248
Callithrix jacchus	T E G P H V G L V Q A S E H P K I E F Y L K N F T S A K D V L F A I K E V G F R G G N S N T G K A L	248
Mus musculus	T E G P H V G L V Q A S E H P K I E F Y L K N F T S A K D V L F A I K E V G F R G G N S N T G K A L	250
Homo sapiens	K H T A Q K F F T V D A G V R K G I P K V V V V F I D G W P S D D I E E A G I V A R E F G V N V F I	298
Callithrix jacchus	K H T A Q K F F T V D A G V R K G I P K V V V V F I D G W P S D D I E E A G I V A R E F G V N V F I	298
Mus musculus	K H T A Q K F F T A D T G V R K G I P K V V V V F I D G W P S D D I E E A G I V A R E F G V N V F I	300
Homo sapiens	V S V A K P I P E E L G M V Q D V T F V D K A V C R N N G F F S Y H M P N W F G T T K Y V K P L V Q	348
Callithrix jacchus	V S V A K P I P E E L G M V Q D V S F V D K A V C R N N G F F S Y H M P N W F G T T K Y V K P L V Q	348
Mus musculus	V S V A K P I P E E L G M V Q D V A F V D K A V C R N N G F F S Y H M P N W F G T T K Y V K P L V Q	350
Homo sapiens	K L C T H E Q M M C S K T C Y N S V N I A F L I D G S S S V G D S N F R L M L E F V S N I A K T F E	398
Callithrix jacchus	K L C T H E Q M M C S K T C Y N S V N I A F L I D G S S S V G D S N F R L M L E F V S N I A K T F E	398
Mus musculus	K L C T H E Q M M C S K T C Y N S V N I A F L I D G S S S V G D S N F R L M L E F V S N I A K T F E	400
Homo sapiens	I S D I G A K I A A V Q F T Y D Q R T E F S F T D Y S T K E N V L A V I R N I R Y M S G G T A T G D	448
Callithrix jacchus	I S D I G A K I A A V Q F T Y D Q R M E F S F T D Y N T K E N V L A V I R N I R Y M S G G T A T G D	448
Mus musculus	I S D I G A K I A A V Q F T Y D Q R T E F S F T D Y N T K E N V L A V L A N I R Y M S G G T A T G D	450
Homo sapiens	A I S F T V R N V F G P I R E S P N K N F L V I V T D G Q S Y D D V Q G P A A A A H D A G I T I F S	498
Callithrix jacchus	A I S F T V R N V F G P I R E S P N K N F L V I V T D G Q S Y D D V Q G P A A A A H D A G I T I F S	498
Mus musculus	A I A F T V R N V F G P I R D S P N K N F L V I V T D G Q S Y D D V R G P A A A A H D A G I T I F S	500
Homo sapiens	V G V A W A P L D D L K D M A S K P K E S H A F F T R E F T G L E P I V S D V I R G I C R D F L E S	548
Callithrix jacchus	V G V A W A P L D D L K D M A S K P K E S H A F F T R E F T G L E P I V S D V I R G I C R D F L E S	548
Mus musculus	V G V A W A P L D D L R D M A S K P K E S H A F F T R E F T G L E P I V S D V I R G I C R D F L E S	550
Homo sapiens	Q Q	550
Callithrix jacchus	Q Q	550
Mus musculus	Q Q	552

Comparisons of amino-acid sequences between human (*Homo sapience*), common marmoset (*Callithrix jaccus*) and mouse (*Mus musculus*)

We compared amino-acid sequences of these three animals and we showed in these six deafness genes amino-acid sequences of common marmoset is more similar to human than mouse. (a) Homology of amino-acid sequences of CX31 was shown. Human vs common marmoset: 95.19%, human vs mouse 82.22% (b) Homology of amino-acid sequences of CRYM was shown. Human vs common marmoset: 99.04%, human vs mouse 88.50% (c) Homology of amino-acid sequences of GRHL2 was shown. Human vs common marmoset: 98.88%, human vs mouse 94.72% (d) Homology of amino-acid sequences of DFNA5 was shown. Human vs common marmoset: 92.14%, human vs mouse 71.37% (e) Homology of amino-acid sequences of ATP6B1 was shown. Human vs common marmoset: 98.44%, human vs mouse 94.72% (f) Homology of amino-acid sequences of COCH was shown. Human vs common marmoset: 98.55%, human vs mouse 93.45%

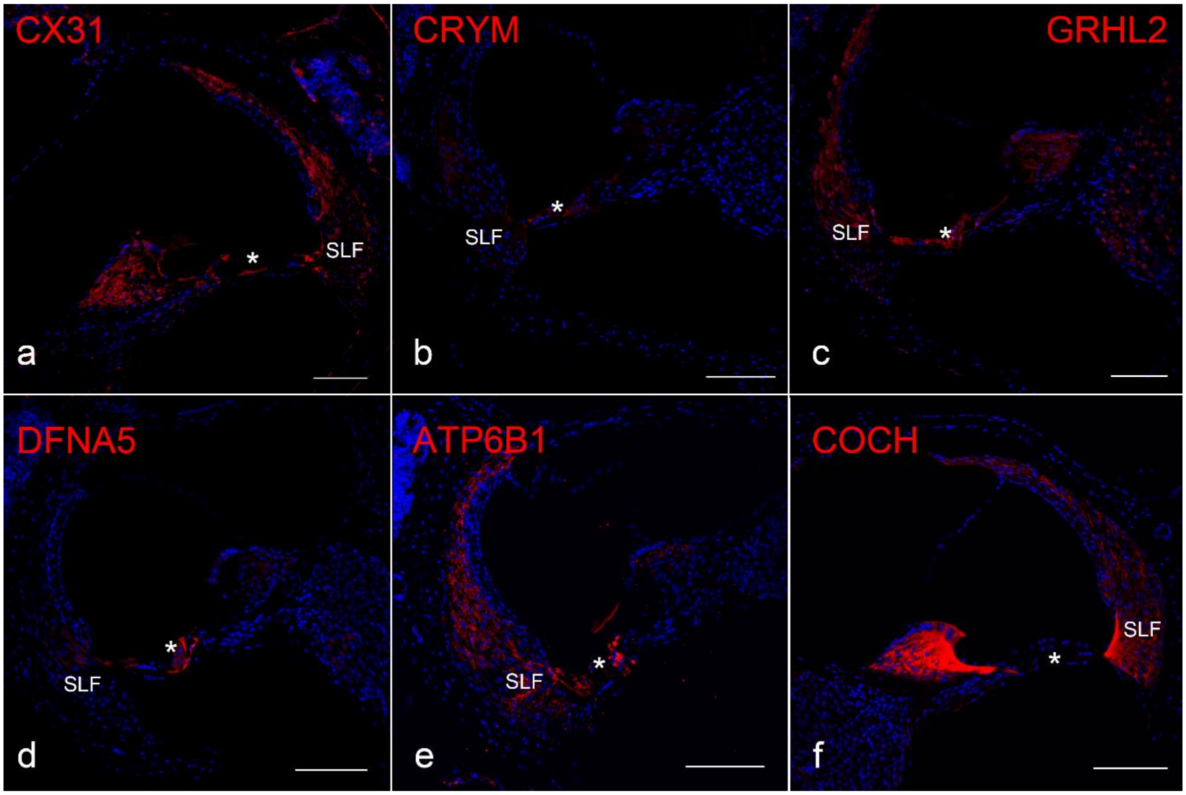

Supplementary Figure. S7

Immunostaining of mouse specimens.

(a) Anti-CX31 antibody recapitulated previous report and strong immunoreactivities were detected in the spiral ligament fibrocytes of mouse and no immunoreactivities was detected in the organ of Corti. (b) Anti-CRYM antibody did not work well against mouse specimens. (c) Anti-GRHL2 antibody used in this report partially recapitulated previous report and immunoreactivities were detected in the organ of Corti. (d) Anti-DFNA5 antibody used in this report partially recapitulated previous report and immunoreactivities were detected in the organ of Corti. (e) Anti-ATP6B1 antibody recapitulated previous report and strong immunoreactivities were detected in the spiral ligament fibrocytes of mouse.

(f) Anti-COCH antibody recapitulated previous report and strong immunoreactivities were detected in the spiral ligament fibrocytes of mouse and no immunoreactivities was detected in the organ of Corti. SLF: Spiral Ligament Fibrocytes, *. Organ of Corti. The nuclei were counterstained with Hoechst (blue). Scale bar: 100 µm.
